# A Cost Analysis of Hospitalizations for Infections Related to Injection Drug Use at a County Safety-Net Hospital in Miami, Florida

**DOI:** 10.1371/journal.pone.0129360

**Published:** 2015-06-15

**Authors:** Hansel Tookes, Chanelle Diaz, Hua Li, Rafi Khalid, Susanne Doblecki-Lewis

**Affiliations:** 1 Department of Internal Medicine, Jackson Memorial Hospital, Miami, Florida, United States of America; 2 Department of Medical Education, University of Miami Miller School of Medicine, Miami, Florida, United States of America; 3 Department of Public Health Sciences, University of Miami Miller School of Medicine, Miami, Florida, United States of America; 4 Department of Jackson Health System Research, Jackson Memorial Hospital, Miami, Florida, United States of America; 5 Department of Internal Medicine, Division of Infectious Diseases, University of Miami Miller School of Medicine, Miami, Florida, United States of America; University of Athens, Medical School, GREECE

## Abstract

**Background:**

Infections related to injection drug use are common. Harm reduction strategies such as syringe exchange programs and skin care clinics aim to prevent these infections in injection drug users (IDUs). Syringe exchange programs are currently prohibited by law in Florida. The goal of this study was to estimate the mortality and cost of injection drug use-related bacterial infections over a 12-month period to the county safety-net hospital in Miami, Florida. Additionally, the prevalence of HIV and hepatitis C virus among this cohort of hospitalized IDUs was estimated.

**Methods and Findings:**

IDUs discharged from Jackson Memorial Hospital were identified using the *International Classification of Diseases*, *Ninth Revision*, codes for illicit drug abuse and endocarditis, bacteremia or sepsis, osteomyelitis and skin and soft tissue infections (SSTIs). 349 IDUs were identified for chart abstraction and 92% were either uninsured or had publicly funded insurance. SSTIs, the most common infection, were reported in 64% of IDUs. HIV seroprevalence was 17%. Seventeen patients (4.9%) died during their hospitalization. The total cost for treatment for injection drug use-related infections to Jackson Memorial Hospital over the 12-month period was $11.4 million.

**Conclusions:**

Injection drug use-related bacterial infections represent a significant morbidity for IDUs in Miami-Dade County and a substantial financial cost to the county hospital. Strategies aimed at reducing risk of infections associated with injection drug use could decrease morbidity and the cost associated with these common, yet preventable infections.

## Introduction

Miami-Dade County ranks first in the United States in new HIV infections [1]. Estimates of HIV prevalence among injection drug users (IDUs) in Miami range from 14% to 23% [2,3]. In 2011 Florida House Bill 7075 was signed into law, giving the State legal authority to close “pill mills” or pain management clinics and enacting stricter medical and pharmacy regulations of prescription opiates starting in July 2012. Heroin-related deaths in Miami increased from 15 to an estimated 36 from 2011 to 2013, and primary treatment admissions for heroin also increased from 227 to 294 during that time [4].

IDUs without access to sterile injection equipment commonly experience skin and soft tissue infections (SSTIs) such as cellulitis and abscesses [5–8]. One study of IDUs in San Francisco found that nearly one-third had experienced SSTIs [7]. SSTIs are associated with inexperience [9], subcutaneous or intramuscular injection known as “skin popping” [7], “speed balling” or the injection of heroin plus cocaine [10,11], and black tar heroin [12,13]. Similarly, the drug of choice may increase the frequency of injection and thereby the risk of infection; for example, IDUs injecting cocaine may inject more than 20 times per day [14,15]. The use of dirty needles and failure to disinfect the skin before injection can also increase the risk of infections [9,10].

In addition to SSTIs, IDUs are frequently hospitalized for complications of bacteremia, which results from inadvertently introducing bacteria into the bloodstream, and can lead to sepsis, endocarditis, and hematogenous osteomyelitis [16,17]. Musculoskeletal infections, such as osteomyelitis, can result from hematogenous spread without a related SSTI [9]. Infective endocarditis is an increasingly common consequence of injection drug use that requires hospitalization for intravenous antibiotics, and in severe cases, cardiac surgery to replace the infected valves [18–20]. Infective endocarditis may be complicated by septic embolization to the lungs, central nervous system, spleen, and other organs [21,22]. These complications can lead to lengthy hospital stays and are associated with a significant mortality risk.

The high incidence of skin and soft tissue infections among IDUs has costly implications for cities with large IDU populations. Studies from urban public hospitals demonstrate that SSTIs are one of the most common reasons for seeking emergency department and inpatient treatment by IDUs, and can comprise a significant proportion of emergency department visits [5,23–25]. Takashi et al. estimated that there were 106,126 hospitalizations for injection drug use-related SSTIs in the US from 1998 to 2001 with an estimated annual nationwide total cost of $193.8 million [26]. The authors found that most IDUs hospitalized with SSTIs were uninsured or relied on Medicare or Medicaid [26]. IDUs with SSTIs tended to have longer and thereby more costly hospital stays compared with other hospitalized patients with an overall cost per hospitalization of $4,449 [26].

This study sought to determine the morbidity, mortality, and cost of hospitalizations for bacterial infections related to injection drug use at the Miami-Dade County safety-net hospital, Jackson Memorial Hospital, over a 12-month period. Additionally, the prevalence of HIV and hepatitis C virus among IDUs admitted to the hospital during the study period was estimated. The data presented will provide a comprehensive estimate of the financial impact of injection drug use-related hospitalizations at a single hospital in Miami-Dade County.

## Methods

### Study design

We conducted a retrospective chart review of patients hospitalized for injection drug use-related infections at the county safety-net hospital, in Miami, Florida during a 12-month period, from July 1, 2013 to June 30, 2014.

### Ethics Statement

The study was approved by the University of Miami Miller School of Medicine Institutional Review Board and the Jackson Health System Clinical Research Review Committee. Informed consent was not obtained from participants and a consent waiver was granted. The data were de-identified prior to analysis.

### Study Setting

Jackson Memorial Hospital is a nonprofit academic medical center and the third largest public hospital in the United States with 1502 licensed beds and an estimated 49,509 annual discharges. It has served as the Miami-Dade County safety-net hospital for nearly a century. The hospital is supported by the taxpayers of Miami-Dade County and governed by the Public Health Trust Board of Trustees.

### Data Collection

We queried the Jackson Memorial Hospital electronic discharge and billing records from July 1, 2013 to June 30, 2014 for injection drug use-related infections in all patients aged 18–65 years. We searched all diagnosis fields in the discharge record database. Discharges included both emergency department discharges and inpatient discharges. Injection drug use-related infection was defined as any discharge over the 12-month period from the inpatient services or emergency department with diagnoses of opiate, cocaine, amphetamine or sedative dependence/abuse and diagnoses of endocarditis, bacteremia/sepsis, osteomyelitis, abscesses or cellulitis. The International Classification of Diseases, Ninth Revision (ICD-9) codes used to identify an injection drug use-related infection are described below. The corresponding medical records were then abstracted for demographic information, length of stay, insurance status, discharge status, and charges related to the services received. HIV and hepatitis C status were also recorded. Specifically, the discharge records for all emergency department visits and inpatient hospitalizations were queried for drug abuse AND infection AND hospitalization between July 1, 2013 and June 30, 2014 AND age 18–65. Drug abuse included opiates OR cocaine OR amphetamines OR sedatives OR other. Infection included endocarditis OR bacteremia/sepsis OR osteomyelitis OR skin/soft tissue infection.

### Definitions

Although the ICD-9 does not explicitly code for injection drug use, we employed an algorithm to identify IDUs using hospital billing records similar to those reported in the literature [5,23,26–28]. Inclusion in the study cohort as a patient with an injection drug use-related infection was based on a combination of ICD-9 codes for illicit drug abuse and medically related infections (Table 1). Patients aged 18–65 were included in the study to increase the specificity of the algorithm. HIV status was queried with ICD-9 codes 042, 079.53, 795.71 and V08. Hepatitis C infection was determined with codes 070.41, 070.44, 070.51, 070.54, V02.62.

**Table 1:** ICD-9 codes used to define injection drug use-related infections.

Diagnoses	ICD-9 codes^a^
Drug abuse diagnoses	
Opiates	E850.0, E850.2, 304.00–304.03, 304.70–304.73, 305.50–305.53, 965.01, 965.09
Cocaine	304.21–304.23, 305.60–305.63, 970.81
Amphetamines	304.41–304.43, 305.71–305.73, 969.72
Sedatives	305.40–305.43
Other	304.60–304.63, 304.80–304.83, 304.90–304.93, 305.90–305.93, 648.33, 648.34
Infections	
Endocarditis	112.81, 421.0, 421.1, 421.9, 424.0–424.3, 424.90, 424.91, 424.99
Bacteremia or Sepsis	038.0, 038.10–038.12, 038.19, 038.2, 038.3, 038.40–038.44, 038.49, 038.8, 038.9, 415.12, 422.92, 449, 785.52, 790.7, 995.90–995.92
Osteomyelitis	730.00–730.29, 730.90–730.99
Skin or soft tissue infections	040.0, 324.0, 324.1, 324.9, 326, 567.22, 567.31, 567.38, 569.5, 572.0, 590.1, 681.00–681.02, 681.10, 681.11, 681.9, 682.0–682.9, 709.8, 728.86, 723.6, 729.30, 729.39, 785.4

^a^Hospitalizations for injection drug use-related infections were defined as having one or more drug abuse diagnoses with one or more infection diagnoses

### Analysis

Descriptive statistics and frequency distributions for demographic, insurance status, infection diagnoses, drug abuse diagnoses, and hospital use variables were generated. Hospital use variables included discharge status and the corresponding charges for each hospitalization. Categorical data were described with numbers and percentages. Continuous variables were reported with median and interquartile range (IQR). A Euler diagram was constructed using EulerAPE version 3.0 to show the relationship between the observed frequencies of infectious diagnoses and respective cost[29]

Hospital charges data were analyzed to evaluate for the potential association of the different types of infections (endocarditis, bacteremia/sepsis, osteomyelitis, and skin and soft tissue infection) with an increased or decreased cost of hospitalization within the cohort. Patients with multiple infections were adjudicated prior to analysis based on the review of their medical records. The principal infection was determined in a hierarchical order based on clinical severity in the following order: endocarditis, bacteremia/sepsis, osteomyelitis, or SSTI. The Wilcoxon rank sums test was used to report a *p* value for the associations between specific infections and the respective total charges using a 2-tailed t test. In comparing adjusted mean charges between the different infection types, several linear regression models were applied with log-transformed outcomes. The models were adjusted for the presence of concomitant infections. To assess the potential impact of insurance status, age, HIV status, and opiate vs. non-opiate drug abuse on the relationship between bacterial infections and charges, the impact of forcing these variables into the multivariable model of charges was evaluated. Adjusted geometric means of the charges and their 95% confidence intervals were calculated. All analyses were completed using SAS Version 9.2 (SAS Institute Inc., Cary, NC).

The charges based on insurance status (county/uninsured, Medicare, Medicaid, private insurance) were reported. The cost over the 12-month period was estimated using the cost-to-charge ratio of 32% for Jackson Memorial Hospital, according to the most recent publicly filed facility profile. [30]

## Results

### Demographics

Demographics are presented in Table 2. Over the 12-month period there were 349 IDUs hospitalized, with a total of 423 admissions at Jackson Memorial Hospital for injection drug-use related infections. Seventy-one percent (n = 248) were male, 52% (n = 180) were white, and 41% (n = 142) were black. The relatively small percentage of Hispanic ethnicity reported in patients in the cohort (6%) compared to the population of Miami-Dade County (65.6%) is due to lack of consistent coding of this category by hospital admitting staff[31]The median age was 47 with an interquartile range (IQR) of 34, 54.

**Table 2:** Demographic characteristics of IDU cohort (N = 349) at Jackson Memorial Hospital.

**Biological sex**	**N**	**(%)**
Male	248	71
Female	101	29
**Race/ethnicity**	**N**	**(%)**
White (non-Hispanic)	180	52
Black (non-Hispanic)	142	41
White Hispanic	17	5
Black Hispanic	5	1
Other/Unknown	5	1
**Age (years)**	**N**	**(%)**
18–29	48	14
30–39	75	21
40–49	81	23
50–59	118	34
60–65	27	8
	**Median**	**IQR**
Age (years)	47	34, 54
**Insurance status**	**N**	**(%)**
County or uninsured	126	36
Medicaid	142	41
Medicare	54	15
Private	27	8

Only 8% (n = 27) of IDUs had private insurance. State-funded Medicaid programs were billed for 41% (n = 142) of patients. Federally-funded Medicare was billed for 15% (n = 54) of patients. Of the IDUs in the cohort, 36% (n = 126) were uninsured. Care for indigent patients at Jackson Memorial Hospital is supported by the taxpayers of Miami-Dade County via a 0.5% sales tax levied since 1991 for the Public Health Trust. Seventeen patients (5%) died during the hospital stay as determined by a discharge status of “expired.”

### Infections and Drug Abuse

**Fig 1:**
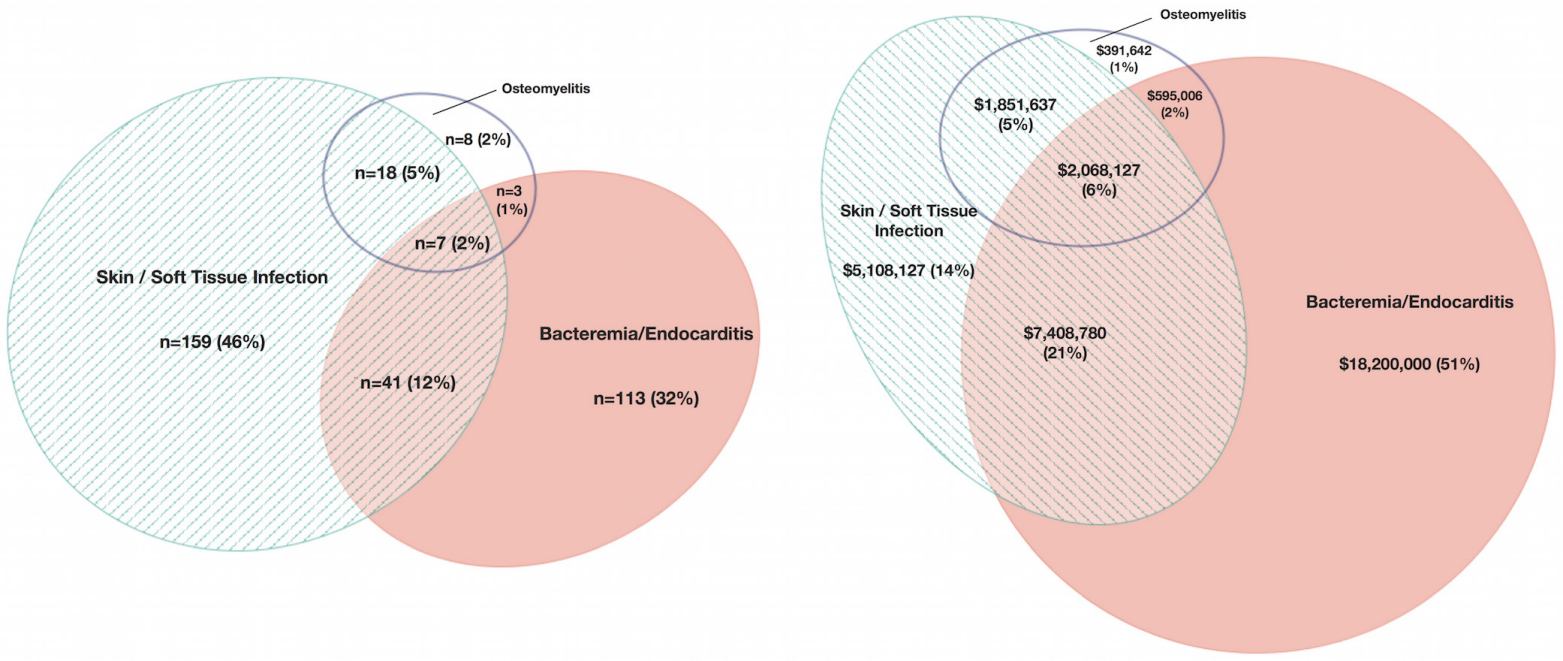
(A) demonstrates the relationship between the observed frequencies of infectious diagnoses. **Euler diagram.** (B) shows the charges per infection type and the proportion of the total charges.

Table 3 presents the frequencies of different infections and drug abuse within the cohort. The majority of the IDUs hospitalized for injection drug use-related infections had SSTIs (64%) resulting from direct inoculation of soft tissues with unsterile injection equipment. Serious infections such as endocarditis and bacteremia/sepsis were reported in 13% and 38% of IDUs, respectively. Osteomyelitis was diagnosed in 10% of the cohort. These infections were not mutually exclusive and 131 of the 349 IDUs in the cohort had multiple infections. Fig 1A shows the overlap of infectious diagnoses within the patients. The seroprevalence of HIV in the IDUs was 17%. Hepatitis C was reported in 15% of patients.

**Table 3:** Infections and Drug Abuse in the IDU cohort at Jackson Memorial Hospital.

**Infections**	**N** ^**a**^	**(%)**
Endocarditis	46	13
Bacteremia or Sepsis	132	38
Osteomyelitis	36	10
Skin and Soft Tissue Infections	225	64
**Seroprevalence**	**N**	**(%)**
HIV	61	17
Hepatitis C Virus	52	15
**Drug Abuse**	**N**	**(%)**
Opiates	129	37
Cocaine	206	59
Amphetamine	11	3
Sedatives	7	2
Other	95	27
**Polysubstance Abuse**	**N**	**(%)**
Yes	82	23
No	267	77

^a^total N for infections >349 due to multiple infection diagnoses in 131 IDUs

Thirty-seven percent of the cohort had opiate abuse diagnosed in discharge records. The majority of IDUs in the study had cocaine abuse diagnosed (59%). Amphetamines and sedatives were less common (3% and 2%, respectively). A substantial proportion of patients had unspecified or other drug abuse reported (27%). Seventy-seven percent of IDUs had only one substance of abuse in their discharge diagnoses.

### Charges for Hospitalizations

Table 4 shows the total charges for IDU-related hospitalizations during the 12-month period and their breakdown per insurance status. The median charge (IQR) for hospitalization for injection drug use-related infection was $39,896 ($14,158-$104,912). The majority of charges were billed to state-funded Medicaid programs ($18,375,845). Miami-Dade County taxpayer funded indigent care was charged $7,094,895 over the 12-month period. Medicare, a federally funded program, was charged $4,702,008. $5,464,512 was billed to private insurers. Accounting for an average cost to charge ratio for Jackson Memorial Hospital, the total cost to the hospital for services rendered for treating injection drug use-related infections from July 1, 2013 to June 30, 2014 was $11,403,923.

**Table 4:** Total charges for hospitalizations of IDUs for infections at Jackson Memorial Hospital.

	**Total**	**Median**	**IQR**	
IDU cohort	$35,637,260	$39,896	$14,158	$104,912
** Total cost**	$11,403,923			
** Insurance Status**	**Total**	**Median**	**IQR**	
County or uninsured	$7,094,895	$18,396	$4,058	$49,987
Medicaid	$18,375,845	$68,209	$24,459	$153,392
Medicare	$4,702,008	$56,872	$27,291	$119,411
Private	$5,464,512	$29,010	$10,580	$100,812

Specific infections were associated with higher charges for hospitalization according to the 2-tailed Wilcoxon Rank Sums test (Table 5). The adjusted mean charge for IDUs with endocarditis was $180,314 versus $71,581 for those without endocarditis (p<0.0001). Likewise, bacteremia/sepsis and osteomyelitis were associated with higher charges (p<0.0001 and p<0.0001, respectively). SSTIs were the only type of infection associated with decreased adjusted mean charges for hospitalization ($100,497 vs $128,432 in IDUs without SSTIs, p<0.0001). When age, HIV status, insurance status and opiate versus non-opiate drug abuse were added to the multivariable model, there was no significant effect on the association between the different types of infection and charges. Charges for the different bacterial infections are shown in Fig 1B.

**Table 5:** Association of infection type with cost after adjusting for concomitant infections.

Infection type	Adjusted Mean^a^ Total Charges	95% Confidence Limits	P-value
Endocarditis	$180,314	$111,238–$292,285	<0.0001
No endocarditis	$71,581	$56,140–$91,270	
Bacteremia or sepsis	$275,642	$187,442–$405,345	<0.0001
No bacteremia or sepsis	$46,826	$34,378–$63,780	
Osteomyelitis	$201,766	$124,302–$327,504	<0.0001
No osteomyelitis	$63,971	$50,573–$80,918	
Skin & soft tissue infection	$100,497	$68,154–$148,188	<0.0001
No skin & soft tissue infection	$128,432	$93,042–$177,284	

^a^Geometric mean adjusted for other infections

## Discussion

This study identified a cohort of 349 IDUs with preventable bacterial infections that resulted in admissions to the county safety net hospital in Miami during a 12-month period. These hospitalizations resulted in $11.4 million in healthcare expenses and 17 deaths. The vast majority of hospitalized IDUs (92%) were either uninsured or relied on publicly funded insurers such as county, state and federal programs. While it is customary for hospitals to bill in excess of expected payment for services rendered based on pre-negotiated reimbursement rates, Florida Medicaid was billed $18.4 million over the study period for injection drug use-related infections. While the number of injection drug use-related overdoses and deaths are one measure of the health impact of drug abuse, this analysis demonstrates that these numbers reflect only a portion of the morbidity, mortality, and cost associated with this high risk behavior.

SSTIs were reported in a majority of the patients in our study. Patients who only had an SSTI had a decreased cost of hospitalization compared with those experiencing other infectious complications, suggesting that earlier diagnosis and treatment of abscesses and cellulitis could lead to cost savings. Studies from urban public hospitals demonstrate that SSTIs are one of the most common reasons for seeking emergency department and inpatient treatment by IDUs, and can burden the system by comprising a significant proportion of ED visits [5,23–25]. Complications of SSTIs such as osteomyelitis, necrotizing fasciitis, and sepsis often require surgical intervention with incision and drainage, debridement, antibiotics and prolonged hospitalization [32–36]. A study conducted at Seattle’s county hospital found that 40% of IDUs who sought ED care for an SSTI were admitted to the hospital. Of those IDUs admitted, 25% required operating room incision and drainage [37]. Similarly, a population-based study found that SSTIs were the leading cause of all non-psychiatric hospital admissions at San Francisco General Hospital from 1999–2000, resulting in $9.9 million in inpatient charges [5]. In response, San Francisco established a skin care clinic and reported that early intervention to prevent SSTIs saved San Francisco General Hospital $8,765,200 in its first year of implementation [38].

Economic modeling of syringe exchange programs for prevention of HIV infection has generally found this strategy to be cost-effective and frequently cost-saving [39–43]. Given the substantial cost of severe bacterial infections related to injection drug use, estimates that consider only the cost and health impact of HIV and hepatitis C infection likely underestimate the potential benefit of these programs. In the 2013 and 2014 Florida Legislative Sessions a bill was presented to establish a pilot syringe exchange program in Miami-Dade County. According to Florida Department of Health projections, the annual cost to operate the syringe exchange program would be $202,451 [44]. With median charges of $39,896 for each member of the cohort, the syringe exchange program would cost less than acute bacterial infections of only 6 IDUs (1.7% of the cohort). Other mitigation strategies, including multidisciplinary methadone treatment and skin care clinics, have demonstrated efficacy in reduction of hospitalization for skin and soft tissue infections at minimal comparative cost [38]. A syringe exchange program in Miami-Dade would also present an opportunity to impact the incidence of HIV in Miami-Dade County, which remains the highest nationwide [1]. In 2014, a bill analysis concluded that if 10% of the HIV infections recorded among IDUs in Miami-Dade County had been prevented, the State of Florida would have saved approximately $124 million [45].

There are several limitations to this study. Because the ICD-9 does not have a specific diagnosis code for injection drug use, the study relied on combinations of ICD-9 codes for drug abuse and infectious consequences, which could have resulted in misclassification bias. The study algorithm is similar to methods previously reported in the literature for the identification of injection drug use-related complications. Additionally, the number of infections associated with drug abuse is likely underestimated due to inconsistent documentation of drug abuse or use of the corresponding codes by physicians and other coding staff. Specific diagnoses such as hepatitis C infection that did not directly relate to the current admission could have been significantly under-reported. Additionally, due to stigma associated with injection drug use, some patients may not have reported drug abuse, leading to further underestimation. Furthermore, this study only reports injection drug use-related hospitalizations at one hospital in Miami-Dade County and likely underestimates the countywide financial burden of these infections. Similar results are likely seen in other hospitals in Miami-Dade County serving the urban poor population. In other cities without syringe exchange programs and a large IDU population, a high economic burden of infections related to injection drug use would be expected at safety net hospitals.

We acknowledge that hospital charges based on insurance status is not equivalent to the cost of care as the hospital negotiates a different average cost-to-charge ratio for each insurer. However, we were able to estimate total cost by using the most recently reported average cost-to charge ratio for Jackson Memorial Hospital.

Despite these limitations, this study adds to recent reports of substantial morbidity, mortality, and expense related to complications of the injection drug use and heroin epidemic in South Florida in the absence of any harm reduction programs [4]. This epidemic is highlighted by a 2012 study that compared the syringe disposal practices of IDUs in Miami to those in San Francisco, where four legal syringe exchange programs operate. The study revealed that Miami had eight times the number of used syringes improperly discarded in public, even though IDUs in San Francisco possessed and therefore disposed of significantly more syringes over a similar period of time [46]. While this study is not a formal cost-effectiveness analysis, costs associated with acute bacterial infections including cellulitis, osteomyelitis, sepsis, and endocarditis are substantial, and prevention of these infections would add to the cost-effectiveness of syringe exchange programs already demonstrated in other modeling studies focusing on HIV prevention. Cities such as Miami may benefit from harm reduction strategies like syringe exchange to reduce the number acute bacterial infections related to injection drug use as well as HIV and hepatitis C infections, and ultimately mitigate the health and economic burdens of these preventable infections.

## Supporting Information

S1 Dataset De-identified Dataset. (XLSX)
